# Exploring the Intersection of E-commerce and Healthcare: A Visual Analysis of Research Trends

**DOI:** 10.7759/cureus.69865

**Published:** 2024-09-21

**Authors:** Alan Zacharia, Ambili Catherine Thomas, Prageesh C Mathew, Regina Sibi Cleetus, Sherin Elizabeth John, Jeena Joseph

**Affiliations:** 1 Department of Economics, St. Thomas College, Thrissur, IND; 2 Department of Commerce, St. Stephen's College, Uzhavoor, IND; 3 Department of Commerce, Newman College, Thodupuzha, IND; 4 Department of Commerce, Mar Ivanios College, Thiruvananthapuram, IND; 5 Department of Commerce, St. George's College, Aruvithura, Kottayam, IND; 6 Department of Computer Applications, Marian College Kuttikkanam (Autonomous), Kuttikkanam, IND

**Keywords:** bibliometric analysis, biblioshiny, e-commerce, health, vosviewer

## Abstract

The role of e-commerce in healthcare is one of the key research areas emerging from the broader digital transformation of health services and consumer interactions. This study presents the bibliometric analysis of literature on e-commerce in healthcare based on publications from 1993 to 2024 in Scopus. In particular, this analysis shows trends in the publication record that have been constantly growing, with prominent increases during the COVID-19 pandemic, now hardwiring the fact that digital technologies are key in promoting global health. The study surveys the thematic evolution of key technologies such as artificial intelligence (AI), which is transforming predictive healthcare analytics; blockchain, which is enhancing security in health data transactions; and telemedicine, which is revolutionizing remote patient care, alongside other emerging technologies that are reshaping global healthcare systems. The research also showcases global collaboration patterns, underlining the international dimensions of scholarly contributions to the area under review. These insights provide a foundation for understanding the current state of e-commerce in healthcare and suggest future directions for research and policy development in this fast-evolving field.

## Introduction and background

E-commerce in healthcare refers to the use of digital platforms and electronic transactions to deliver healthcare services, products, and information. This includes the online sale of pharmaceuticals, telemedicine services, electronic health records management, and the use of digital tools for patient interaction, such as appointment booking, virtual consultations, and health monitoring applications. Within this context, e-commerce in healthcare serves as a transformative approach, enabling patients and providers to engage in more efficient, convenient, and cost-effective exchanges, particularly as the demand for digital health solutions grows in the wake of technological advances and global challenges like the COVID-19 pandemic. The fusion of e-commerce and healthcare is an emerging area that applies to digital technologies to improve delivery and healthcare access while ultimately seeking to control spiraling costs. This review of related literature deals with various perspectives of healthcare e-commerce that deal with the acceptance, usage, and customer satisfaction levels as well as the issues and opportunities it offers. The last two years have seen much e-commerce activity in the health sector through hospitals and health facilities. A narrative review by Ardalan and Mirzaei has elaborated that the willingness to accept e-commerce in health organizations was affected due to the availability of required hardware and software with managerial support and following benefits regarding related quality of services and financial performance [1].

The rapid rise of information technology has influenced consumer behavior with regard to acceptance and use of a variety of services, including healthcare services. A study by Aishwarya et al. surveyed the real usage and behavioral intention to use e-healthcare services among Indian consumers. In this regard, it is worthwhile to mention that the rapid growth in mobile health applications and other associated digital channels has influenced consumer behavior to a great extent; the necessity for e-healthcare services in modern times is inevitable [2]. Customer satisfaction is a very important area in which e-commerce in healthcare plays a vital role. Chatterjee et al. did a study using text mining and machine learning to analyze customer satisfaction against several subcategories of healthcare e-commerce. The findings revealed that customer satisfaction lay on aspects of the service core coupled with the elicited emotions from the experience of the service. This finding supports the fact that an e-commerce site must be designed to be both functionally and emotionally engaging for the user [3].

Another significant trend in the health sector is the development of trans-border e-commerce platforms. According to Song, such interactive platforms are fast becoming very crucial in the sales and marketing of health products and services across borders, with data analytics and artificial intelligence at the center. These not only offer a wide range of opportunities for innovation but also present challenges related to regulatory compliance and data security [4]. Digitization of healthcare products in China allows democratization through e-commerce. Antwi et al. provided a systematic review that highlights how e-commerce enables a restructured healthcare delivery system and facilitates personal and informed decision-making by consumers. This will characterize a new era in healthcare consumerism, where patients expect a retail-like experience in their interactions with healthcare [5].

Another growing area of focus is the role of e-commerce in promoting sustainability within healthcare. Through the COVID-19 pandemic, e-commerce played a significant role in holding up the supply chain and meeting public health demands; however, to attain long-term sustainability, there exists a dimension wherein economic, social, and environmental dimensions have to be harmoniously balanced. This view comes out through studies using big data analytics, underlining the necessity for sustainable e-commerce practices toward these three pillars [6]. The increase in e-commerce in healthcare also brings out challenges to ensure consumer safety. Attjioui et al. pointed out that the rapid growth of online sales of health products has entailed convenience but also increased risks related to the proliferation of falsified medicines. It underlines the fact that there is an immediately felt need for robust regulatory frameworks addressing these risks and protecting consumers from them [7].

Bibliometric analysis is an important and effective quantitative tool that can be applied in the analysis of trends in publications in literature [8-10]. In its capacity to comprehensively review publications, citations, as well as other scholarly outputs, it makes research easy to recognize emerging patterns, emphasize key works, and delineate the intellectual structure underpinning a given discipline [11-17]. Biblioshiny (Naples, Italy: University of Naples Federico II) is a web-based, user-friendly interface for the R-based bibliometric package that offers advanced possibilities for in-depth analysis and visualization without extensive programming knowledge [18-25]. The other important tool often used to build and analyze bibliometric networks like co-authorship, co-citation, and keyword co-occurrence, which can draw meaningful insights by its visual representation of complex research areas, is VOSviewer (Leiden, Netherlands: Leiden University) [26-31].

This study aimed to provide a comprehensive bibliometric analysis of e-commerce in healthcare, from 1993 to 2024. Specifically, the study seeks to identify and analyze major themes and rising topics within this interdisciplinary field, focusing on key trends and the technological evolution of e-commerce in healthcare. Additionally, this study aimed to examine global collaboration patterns, highlighting the most influential contributors and their impact on the development of the field. By analyzing the role of emerging technologies such as artificial intelligence, blockchain, and telemedicine, the study seeks to provide a deeper understanding of how these advancements are shaping healthcare delivery. Finally, the study offers insights into future research directions and policy developments, with particular attention to the digital transformation accelerated by the COVID-19 pandemic.

## Review

Materials and methods

The Scopus database was selected as the primary source for bibliographic data due to its extensive coverage of peer-reviewed literature across multiple disciplines, providing access to high-quality journals. The search strategy employed a Boolean query as follows: "E-commerce" OR "Electronic commerce" AND "health", aimed at capturing documents that included variations of the term "e-commerce" or "electronic commerce" in combination with "health." No language filters were applied, ensuring the inclusion of studies from various linguistic backgrounds. The search was restricted to the time frame between 1993 and 2024 to capture the evolution of research in e-commerce and healthcare. This strategy allowed for a comprehensive view of the developments in this field over a significant period.

**Figure 1:**
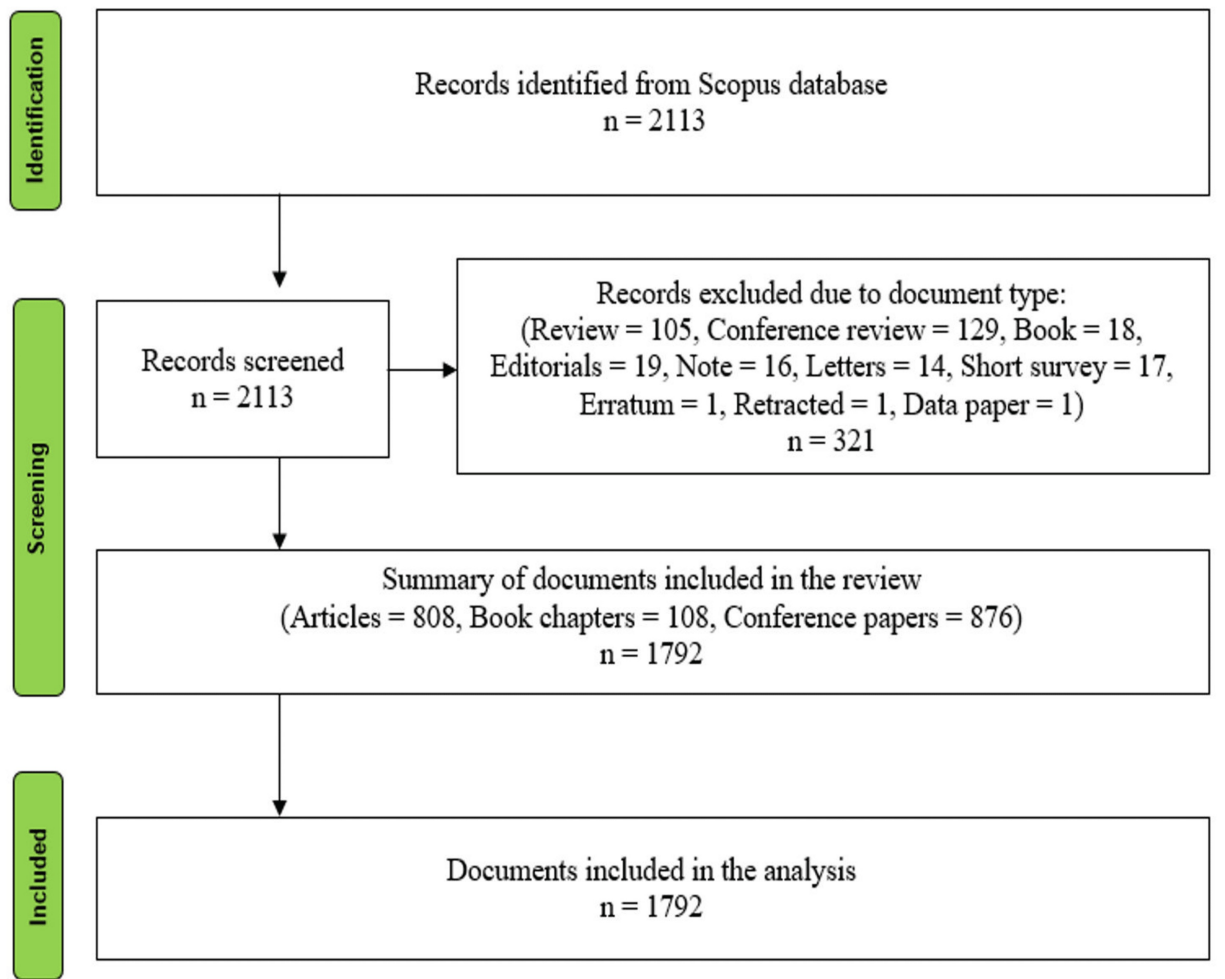
PRISMA flow diagram of the literature selection process for e-commerce in healthcare studies. PRISMA: Preferred Reporting Items for Systematic Reviews and Meta-Analyses

The Preferred Reporting Items for Systematic Reviews and Meta-Analyses (PRISMA) methodology was applied to refine the dataset, following a structured three-stage process. In the first stage, relevant documents were identified based on the search query. In the second stage, documents such as reviews, editorials, letters, notes, and short surveys were excluded to ensure the analysis focused on research-intensive publications. Only journal articles, book chapters, and conference papers were retained for analysis, as these document types provide substantive contributions to the field. Specific inclusion criteria were applied to ensure that the selected documents focused on the integration or application of e-commerce in healthcare. Exclusion criteria filtered out documents that were too general or did not provide significant insights into e-commerce’s role in healthcare. As a result, 1,792 documents from 1,073 sources were included in the final dataset, which was exported in CSV format for subsequent analysis. Figure 1 depicts the PRISMA flow diagram outlining this process. Bibliometric analysis was conducted using VOSviewer and Biblioshiny, two tools commonly used for building and analyzing bibliometric networks, including co-authorship, co-citation, and keyword co-occurrence. VOSviewer was employed for its ability to visualize collaboration patterns and thematic relationships, while Biblioshiny was selected for its user-friendly interface and advanced trend analysis capabilities, offering complementary insights into research themes and technological advancements in e-commerce and healthcare.

Table 1 provides a detailed summary of the key findings from the analysis of e-commerce in healthcare research. The data spans from 1993 to 2024, encompassing 1,792 documents sourced from 1,073 different journals, books, and other publications. The analysis reveals a stable annual growth rate, with an average document age of 8.9 years and an average of 12.91 citations per document. The dataset includes 53,787 references, highlighting the extensive research foundation in this area. In terms of content, the study identified 9,744 "Keywords Plus" (ID) and 4,898 author-provided keywords (DE). A total of 4,738 authors contributed to the field, with 274 authors producing single-authored documents. Collaboration among researchers is evident, with 3.1 co-authors per document and 16.85% of publications involving international co-authorships. The types of documents analyzed include 808 articles, 108 book chapters, and 876 conference papers, showcasing a diverse range of scholarly contributions to the intersection of e-commerce and healthcare.

**Table 1:** Summary of key information and characteristics of studies identified in the bibliometric analysis of e-commerce in healthcare.

Description	Results
Main information about the data	
Timespan	1993 to 2024
Sources (journals, books, etc.)	1,073
Documents	1,792
Annual growth rate %	0
Document average age	8.9
Average citations per doc	12.91
References	53,787
Document contents	
Keywords Plus (ID)	9,744
Author's Keywords (DE)	4,898
Authors	
Total authors	4,738
Authors of single-authored docs	274
Authors collaboration	
Single-authored docs	293
Co-authors per doc	3.1
International co-authorships %	16.85
Document types	
Article	808
Book chapter	108
Conference paper	876

Annual Scientific Production

**Figure 2:**
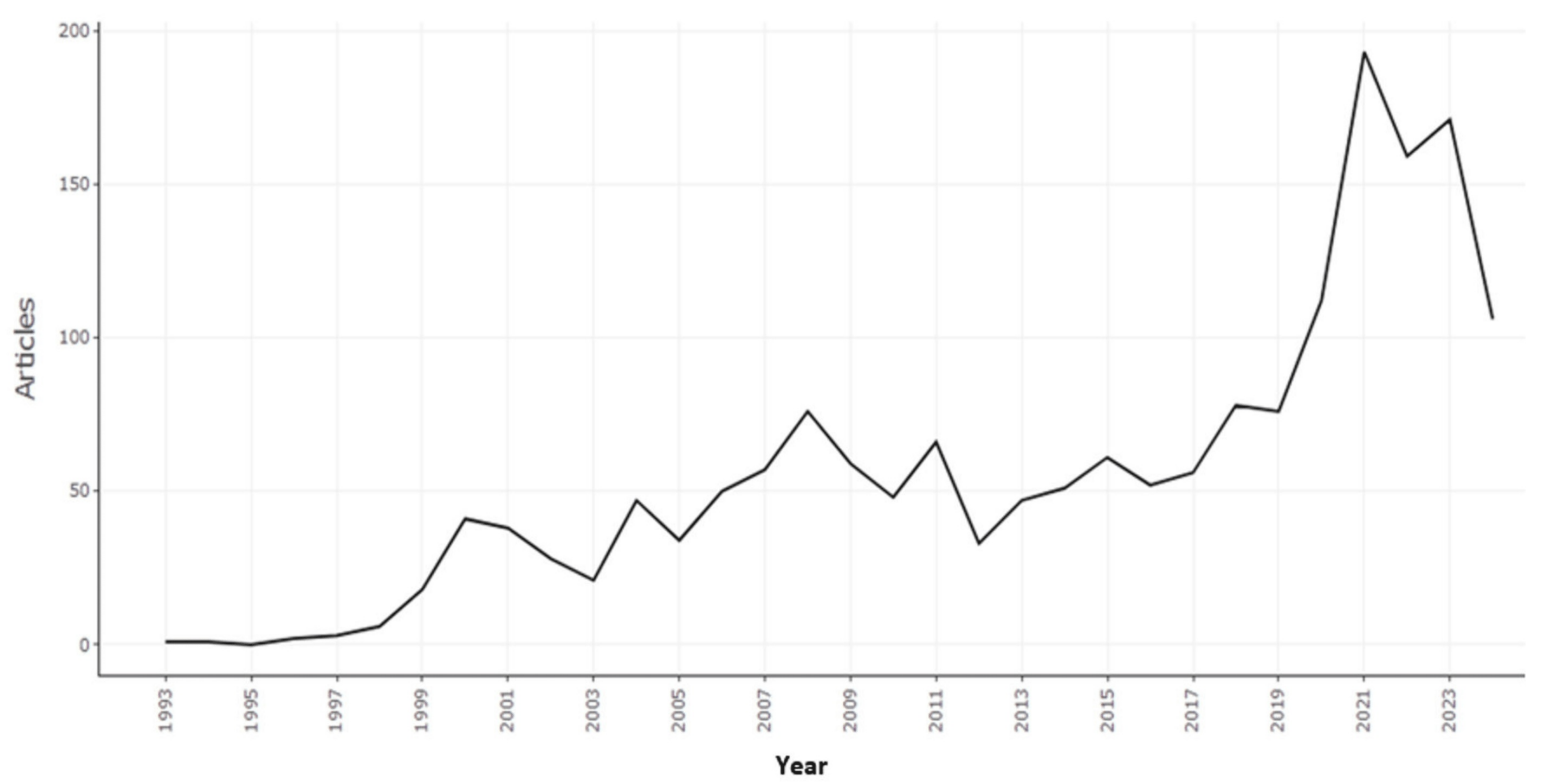
Annual scientific production of publications on e-commerce in healthcare (1993-2024). This figure is created by the authors of this study using the tools Biblioshiny and VOSviewer.

Figure 2 illustrates the annual scientific production in the field of e-commerce in healthcare from 1993 to 2024. The chart shows a gradual increase in the number of articles published over the years, with a noticeable spike in production starting around 2019. The peak appears to have occurred around 2021, followed by a slight decline in the subsequent years. This trend reflects the growing interest and research activity in the intersection of e-commerce and healthcare, particularly in the years leading up to and following the COVID-19 pandemic, which likely influenced the surge in publications during that period.

Most Relevant Authors

Table 2 highlights the most prolific authors in the field of e-commerce in healthcare, with a focus on the number of articles each has contributed. Liu Y, Zhang X, and Zhang Y lead the list, each with 13 articles, demonstrating their significant influence in this area of research. Li Y follows closely with 11 articles, while Li J and Yee G each have contributed 10 articles. Liu L, Wickramasinghe N, and Yang X have each authored nine articles, showcasing their active involvement in the field. Chen Y, with eight articles, rounds out the list of the top contributors. The prominence of these authors indicates their central role in advancing research on the intersection of e-commerce and healthcare, likely providing foundational insights and driving ongoing scholarly discussions in this rapidly evolving domain.

**Table 2:** Most prolific authors in e-commerce in healthcare research based on publication count.

Authors	No. of articles
Liu Y	13
Zhang X	13
Zhang Y	13
Li Y	11
Li J	10
Yee G	10
Liu L	9
Wickramasinghe N	9
Yang X	9
Chen Y	8

Most Relevant Sources

Table 3 presents the most relevant sources for publications in the field of e-commerce in healthcare, based on the number of articles published. The Lecture Notes in Computer Science (including its subseries in Artificial Intelligence and Bioinformatics) is the leading source, with 69 articles, indicating its prominence in disseminating research in this domain. The Proceedings of the International Conference on Electronic Business (ICEB) follow closely with 61 articles, reflecting its role as a key venue for presenting cutting-edge research in electronic business applications within healthcare. The ACM International Conference Proceeding Series has published 55 articles, further highlighting the importance of conference proceedings in this field. Other significant sources include the Lecture Notes in Networks and Systems with 26 articles, and the IFIP Advances in Information and Communication Technology with 20 articles, both of which contribute to the interdisciplinary exchange of knowledge. Additionally, the Advances in Intelligent Systems and Computing and the International Journal of Environmental Research and Public Health, each with 16 articles, and Studies in Health Technology and Informatics with 14 articles, underscore the diversity of sources contributing to the research landscape of e-commerce in healthcare. These sources collectively represent a broad spectrum of research outputs, encompassing both conference proceedings and journal articles, and play a critical role in shaping the discourse in this field.

**Table 3:** Most influential sources in e-commerce in healthcare research.

Sources	No. of articles
Lecture Notes in Computer Science (Including Subseries Lecture Notes in Artificial Intelligence And Lecture Notes In Bioinformatics)	69
Proceedings of the International Conference on Electronic Business (ICEB)	61
ACM International Conference Proceeding Series	55
Lecture Notes in Networks and Systems	26
IFIP Advances in Information and Communication Technology	20
Advances in Intelligent Systems and Computing	16
International Journal of Environmental Research and Public Health	16
Studies in Health Technology and Informatics	14

Countries' Scientific Production

Table 4 provides an overview of the scientific production by country in the field of e-commerce in healthcare. China leads with a significant contribution of 1,030 articles, indicating its dominant role in advancing research in this area. The United States follows with 733 articles, showcasing its strong presence and continued investment in healthcare and e-commerce research. India ranks third with 615 articles, reflecting its growing focus on integrating digital technologies in healthcare. The United Kingdom, with 222 articles, and Australia, with 191 articles, also demonstrate substantial research activity in this field.

**Table 4:** Countries' scientific production in e-commerce in healthcare research based on the number of publications.

Country	No. of articles
China	1,030
USA	733
India	615
UK	222
Australia	191
Italy	187
Spain	170
Canada	162
Germany	146
Indonesia	126

Italy, Spain, Canada, Germany, and Indonesia contribute 187, 170, 162, 146, and 126 articles, respectively, highlighting the global nature of research in e-commerce in healthcare. These countries represent a diverse range of regions, indicating widespread interest and scholarly contributions across different healthcare systems and technological landscapes. The distribution of articles across these countries underscores the international collaboration and the varying levels of emphasis on e-commerce applications in healthcare around the world.

Trend Topics

**Figure 3:**
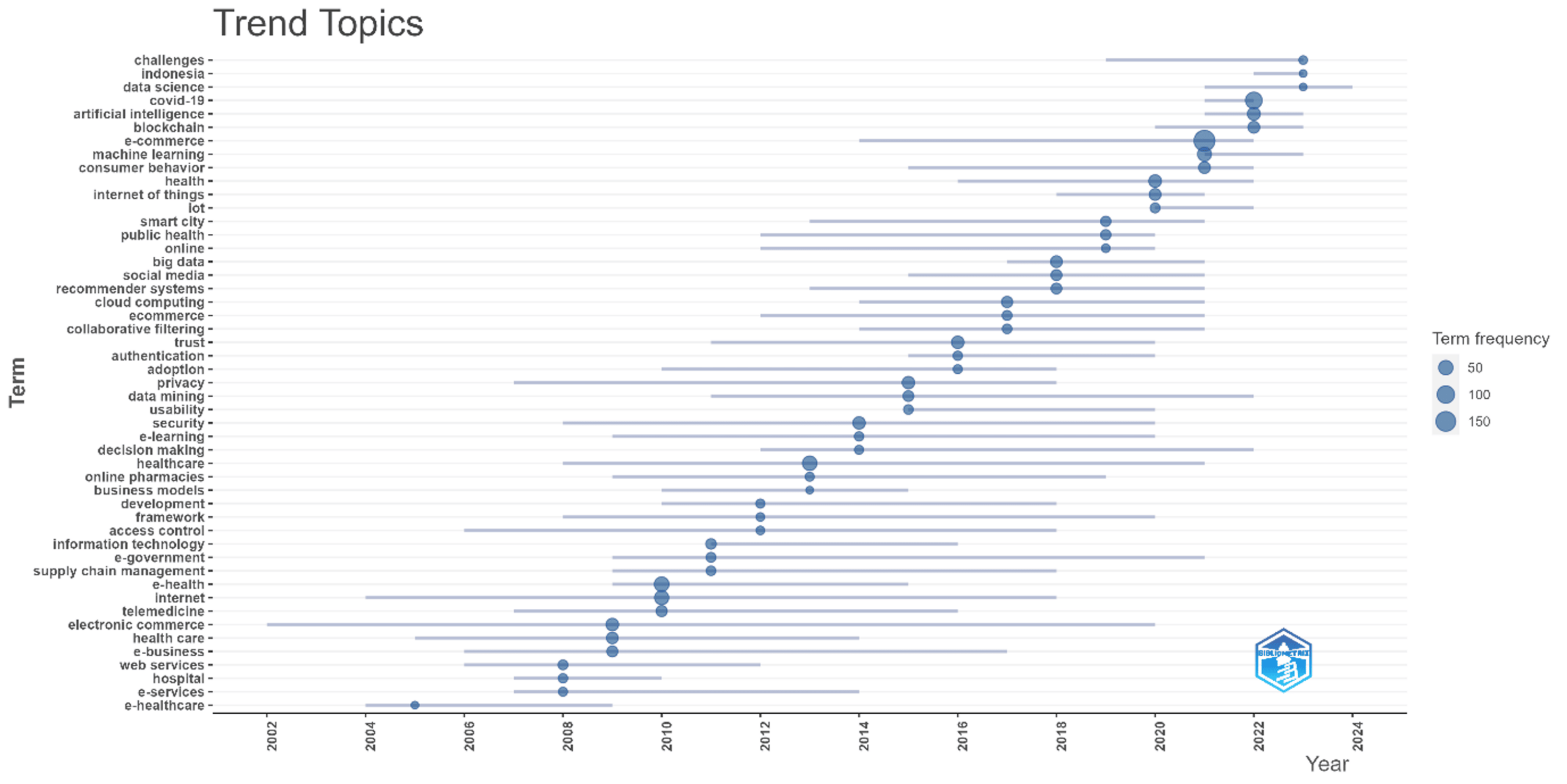
Trending topics in e-commerce in healthcare research. This figure is created by the authors of this study using the tools Biblioshiny and VOSviewer.

Figure 3 illustrates the trend topics in e-commerce and healthcare research over time, highlighting the frequency and evolution of key terms used in the literature from 2002 to 2024. The size of the circles corresponds to the term frequency, with larger circles indicating more frequent usage. Early in the timeline, terms like "e-healthcare," "e-services," and "telemedicine" were prominent, reflecting the initial focus on integrating digital solutions into healthcare. As the years progressed, newer topics such as "cloud computing," "big data," "social media," and "machine learning" began to emerge, signifying the growing importance of advanced technologies and data-driven approaches in healthcare e-commerce. In more recent years, terms like "blockchain," "artificial intelligence," and "COVID-19" have gained prominence, indicating a shift towards secure, intelligent, and pandemic-responsive solutions. The appearance of terms like "data science," "IoT," and "smart city" also points to the integration of e-commerce in broader digital health ecosystems. Overall, the trend topics depicted in Figure 3 provide a visual summary of the evolving research focus within the intersection of e-commerce and healthcare, showcasing how technological advancements and global events have shaped the discourse over time.

Thematic Map

**Figure 4:**
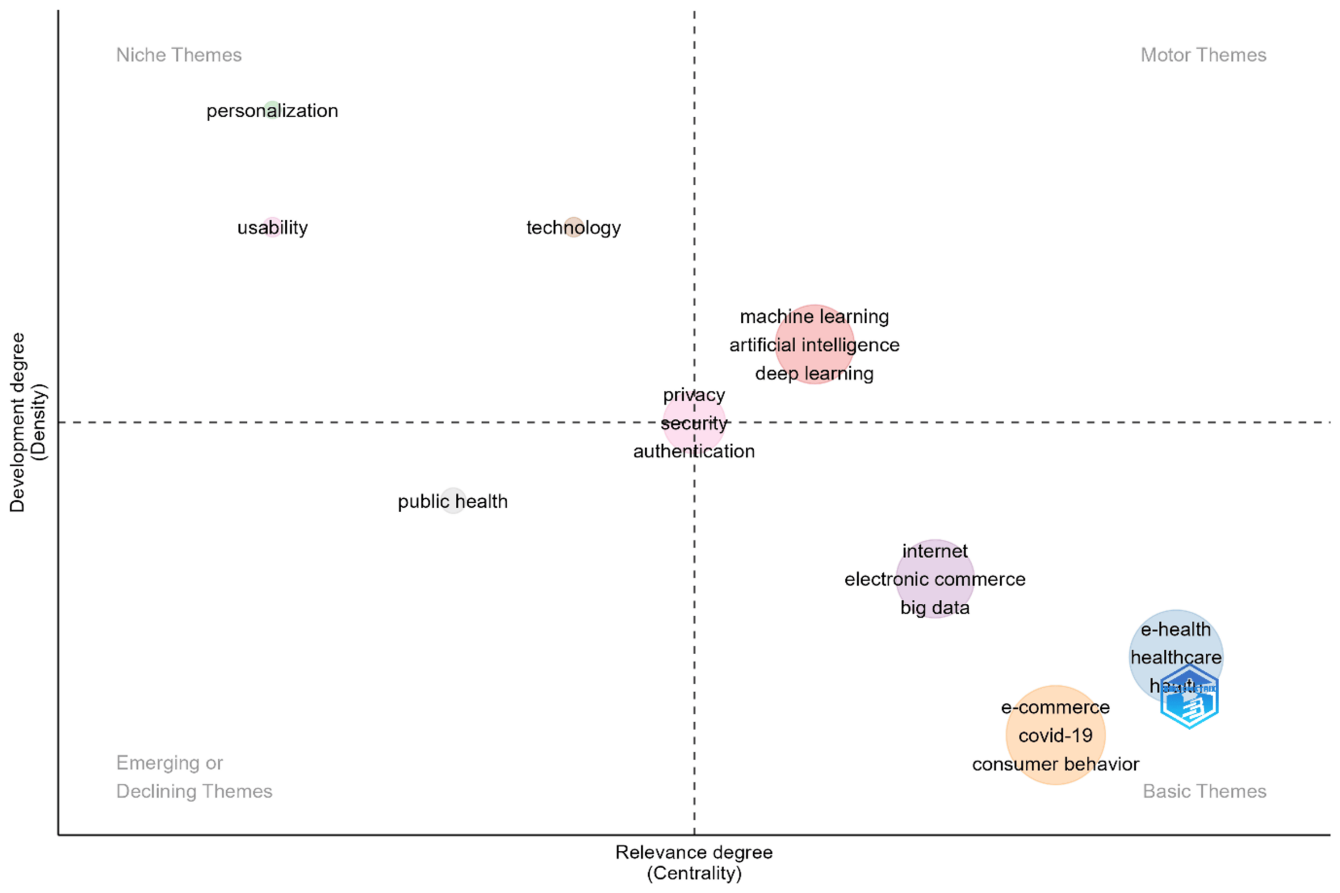
Thematic map of key research themes in e-commerce in healthcare. Node size and color: the size of each node represents the frequency of occurrence of the topic in the literature, with larger nodes indicating more frequently studied topics. The color of the nodes indicates the density of the research on that topic, with darker colors representing higher density, implying that the topic is more internally coherent and well-developed. This figure is created by the authors of this study using the tools Biblioshiny and VOSviewer.

Figure 4 provides a thematic map of research topics within the intersection of e-commerce and healthcare, categorizing these topics based on their development degree (density) and relevance degree (centrality). The map is divided into four quadrants, each representing different types of themes. In the niche themes quadrant (top-left), topics such as "personalization" and "usability" are highly developed but have lower centrality, indicating they are specialized areas within the field that, while well-explored, are less connected to the core themes. The motor themes quadrant (top-right) includes highly relevant and well-developed themes like "machine learning," "artificial intelligence," and "deep learning," which are central driving forces in advancing research in e-commerce and healthcare. The basic themes quadrant (bottom-right) features foundational topics such as "e-health," "healthcare," "e-commerce," "COVID-19," and "consumer behavior," which are highly relevant to the field but less developed, indicating areas that form the core of the research landscape but may need further exploration. Lastly, the emerging or declining themes quadrant (bottom-left) includes topics like "public health," which are either growing in importance or becoming less significant, as indicated by their lower centrality and development. This thematic map offers a comprehensive view of the research landscape, illustrating the relationship between the development and relevance of various topics within e-commerce and healthcare.

Co-authorship Between Countries

**Figure 5:**
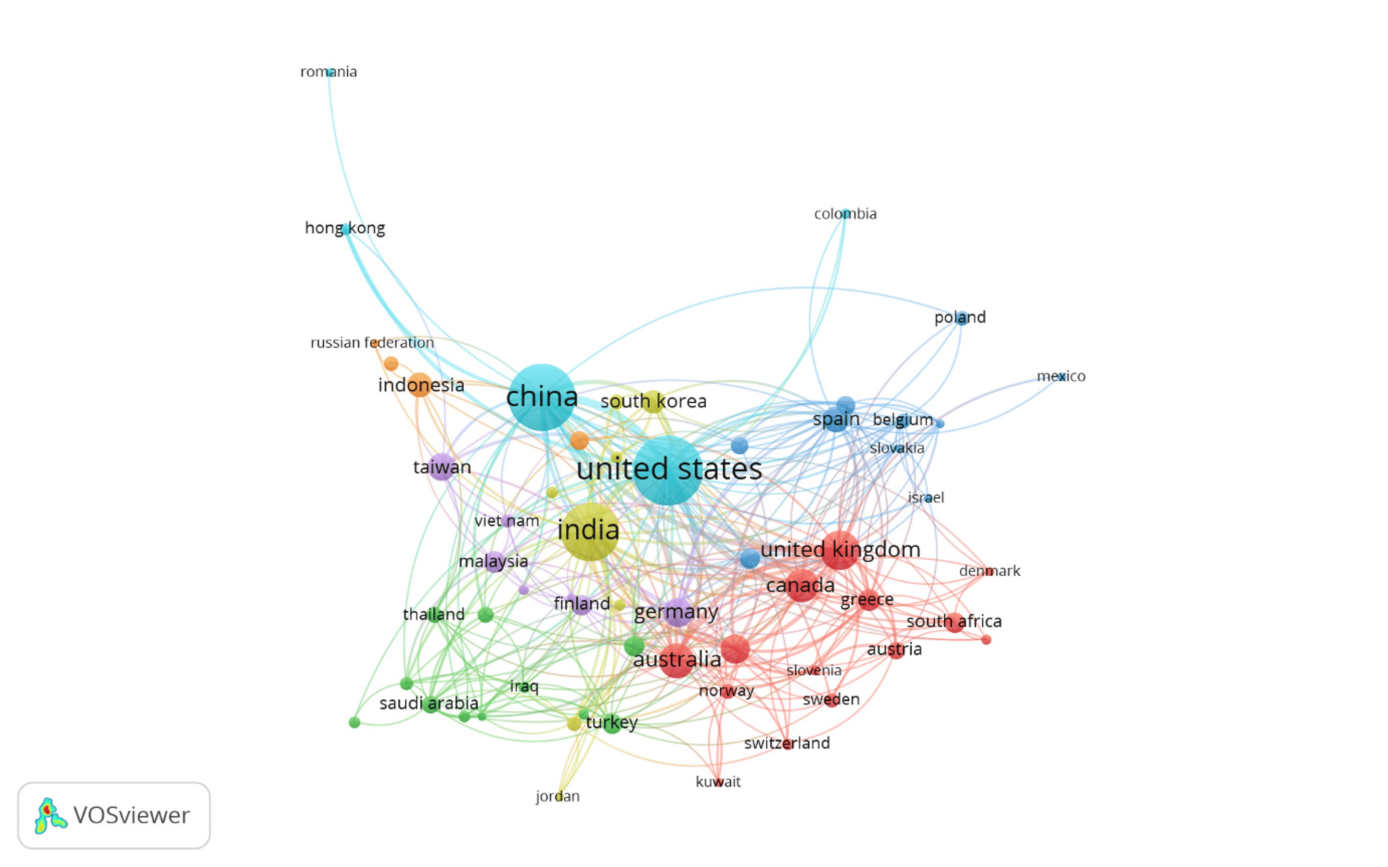
Network visualization of co-authorship between countries in e-commerce in healthcare research. Node size: the size of each node represents the number of publications contributed by each country. Larger nodes indicate countries with a higher volume of publications in this research area. Node color: the color of each node represents different clusters of countries that frequently collaborate with each other. Each color indicates a distinct collaboration cluster. Thickness of connecting lines: the thickness of the lines connecting the nodes indicates the strength of the co-authorship links between countries. Thicker lines represent stronger or more frequent collaboration between the countries. This figure is created by the authors of this study using the tools Biblioshiny and VOSviewer.

Figure 5 illustrates the co-authorship network between countries in e-commerce and healthcare research. The size of each node corresponds to the volume of publications from each country, with larger nodes indicating higher numbers of published works. The thickness of the connecting lines represents the strength of collaborative relationships between countries, where thicker lines indicate more frequent or stronger co-authorship ties. The colors of the nodes represent distinct clusters or groups of countries that frequently collaborate with one another. Each color signifies a different collaboration group, highlighting regional or thematic research networks. For instance, countries like China, the United States (US or USA), and India are central in their respective clusters, indicating their leadership and extensive involvement in international research partnerships. China's central position, with connections to countries such as South Korea and Indonesia, underscores its active role in fostering international collaborations, particularly within Asia. The United States shows strong ties with the United Kingdom, Canada, and other European countries, reflecting its significant involvement in Western research networks. Meanwhile, India demonstrates strong connections within Asia and beyond, reflecting its growing prominence in global healthcare e-commerce research.

Co-occurrence of Keywords

**Figure 6:**
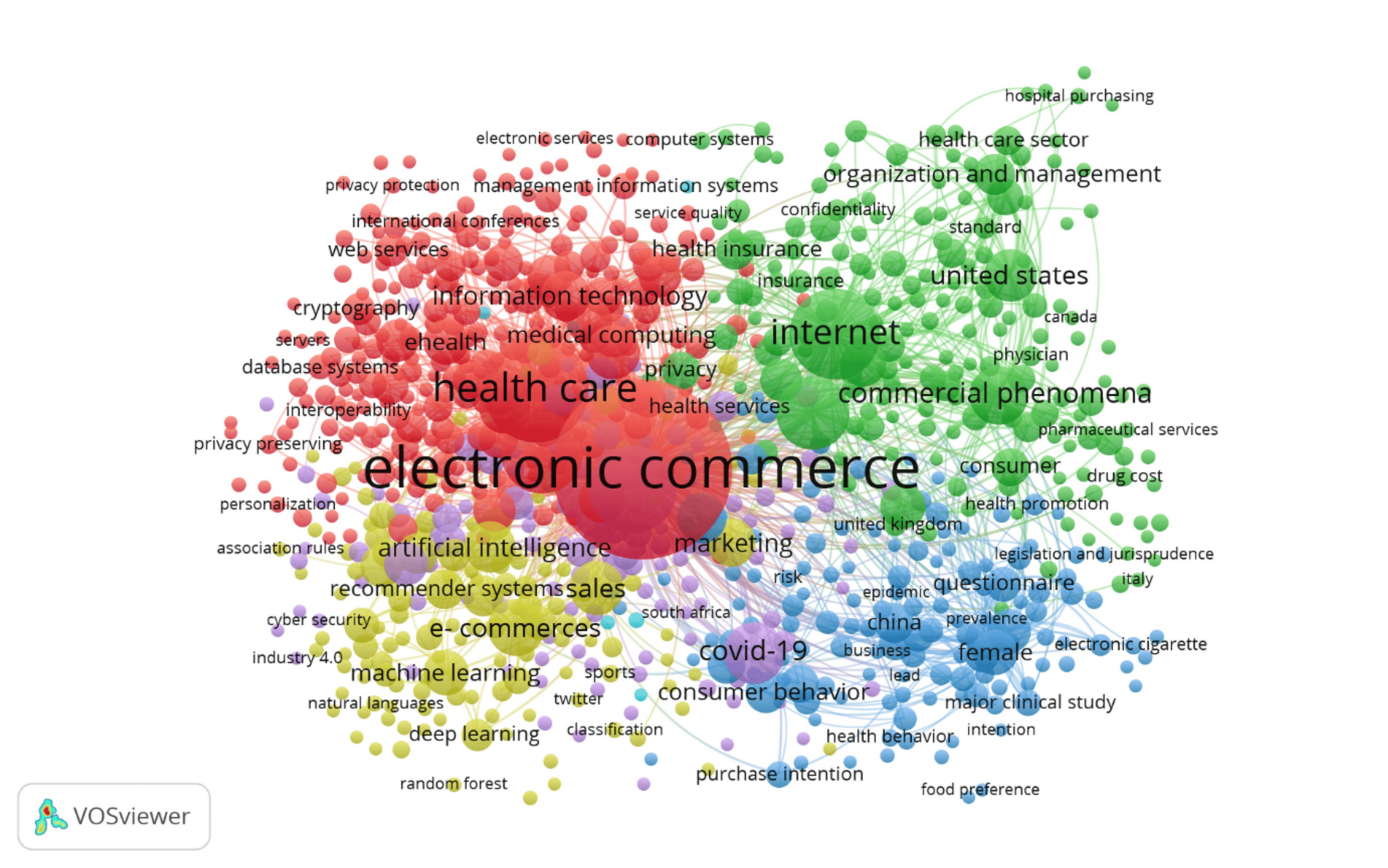
Network visualization of keyword co-occurrence in e-commerce in healthcare research. Node size: the size of each node represents the frequency of occurrence of the keyword in the literature. Larger nodes indicate keywords that are more frequently used in the research articles. Node color: the color of each node corresponds to different clusters of related keywords. Each color represents a distinct thematic area or research focus within the broader field. Thickness of connecting lines: the thickness of the lines connecting the nodes represents the strength of the association between the keywords. Thicker lines indicate a higher frequency of co-occurrence between the connected keywords. This figure is created by the authors of this study using the tools Biblioshiny and VOSviewer.

Figure 6 visualizes the co-occurrence network of keywords in e-commerce and healthcare research, with the size of each node representing the frequency of keyword usage and the thickness of connecting lines indicating the strength of relationships between the terms. The different colors in the network represent thematic clusters, each highlighting distinct areas of research within this interdisciplinary field. The red cluster, which centers around healthcare systems and technologies, includes prominent keywords such as "health care," "medical computing," and "electronic commerce." This cluster signifies the foundational focus on integrating digital commerce with healthcare services. Terms like "information technology" and "e-health" within this cluster further emphasize the technological advancements driving this transformation. This cluster is significant because it represents the core technological backbone of the field, showing how e-commerce platforms are applied directly to healthcare service delivery. The green cluster is focused on organizational and management aspects of healthcare e-commerce. Keywords such as "organization and management," "commercial phenomena," and "internet" reflect research into how healthcare organizations are adopting digital platforms to manage services and products. Terms like "health insurance" and "confidentiality" illustrate the importance of managing sensitive patient data and ensuring secure transactions in digital health environments. This cluster is critical as it highlights the managerial challenges and solutions in implementing e-commerce in healthcare systems. The yellow cluster revolves around artificial intelligence (AI) and machine learning applications. Central terms such as "artificial intelligence," "machine learning," and "deep learning" underscore the increasing reliance on AI-driven tools for enhancing healthcare e-commerce, particularly in predictive analytics and personalized medicine. This cluster is important because it showcases how advanced technologies are driving innovation in healthcare, opening new avenues for personalized and data-driven care delivery. The blue cluster focuses on consumer behavior, featuring keywords such as "consumer behavior," "COVID-19," and "purchase intention." This cluster reflects research on how consumers interact with healthcare e-commerce platforms, particularly during the global pandemic. Terms like "questionnaire" and "health behavior" show an interest in understanding patient attitudes and preferences, which are essential for designing more effective digital healthcare services. This cluster is significant as it highlights the importance of consumer engagement and response to healthcare e-commerce, an area that has grown rapidly due to the COVID-19 pandemic. Overall, this co-occurrence network reveals the interconnectedness of healthcare, technology, and consumer behavior in driving research within the e-commerce healthcare domain. Each cluster plays a crucial role in shaping different aspects of this interdisciplinary field, from technological innovation and AI applications to managerial solutions and consumer-focused studies.

Bibliographic Coupling Between Countries

**Figure 7:**
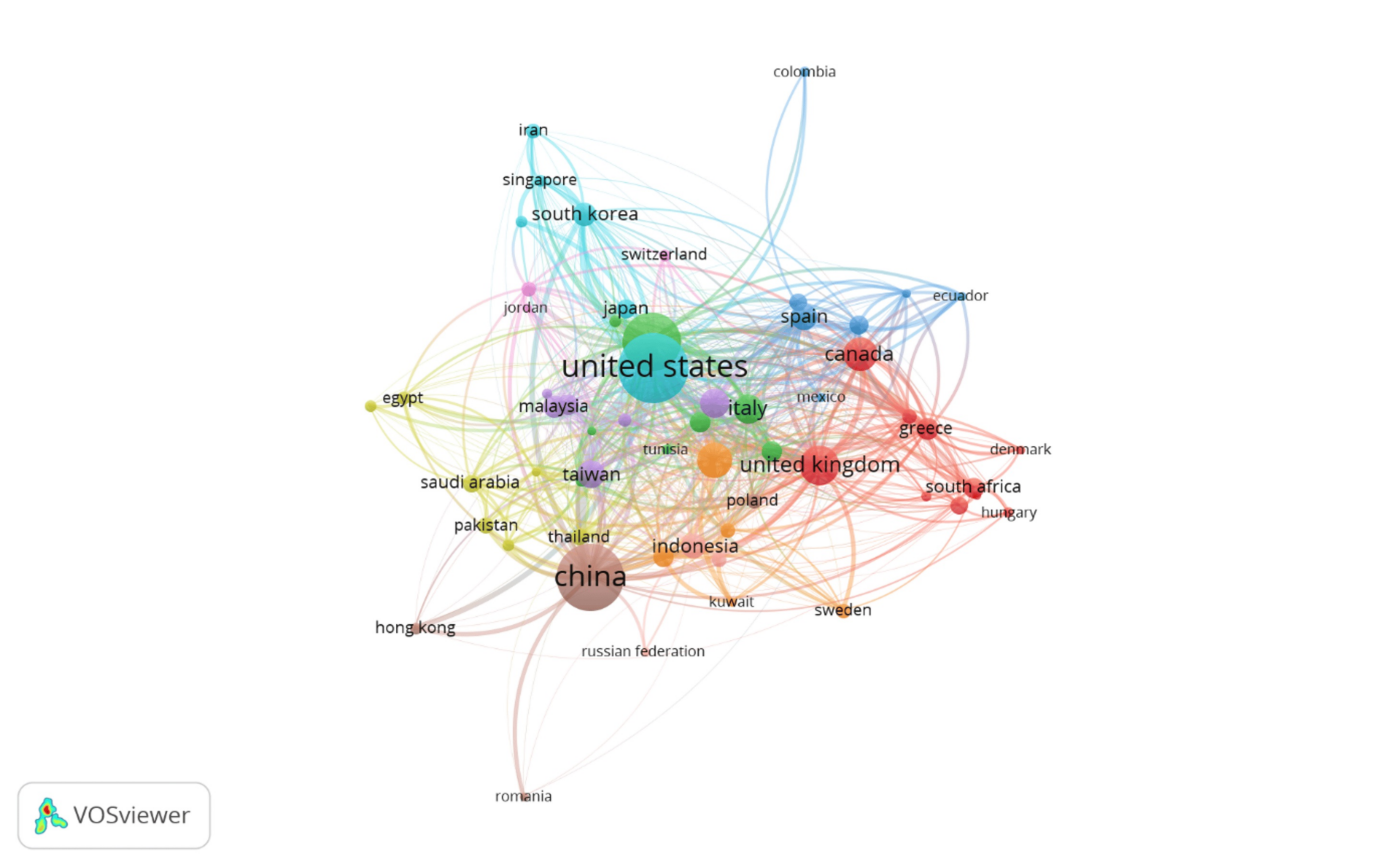
Network visualization of bibliographic coupling among countries in e-commerce in healthcare research. Node size: the size of each node represents the number of publications from each country that are coupled based on shared references. Larger nodes indicate countries with a higher volume of coupled publications, signifying greater influence or contribution to the research network. Node color: the color of each node represents different clusters of countries that are closely linked through bibliographic coupling. Each color corresponds to a specific group of countries that frequently reference similar bodies of literature, indicating shared research interests or collaborations. Thickness of connecting lines: the thickness of the lines connecting the nodes indicates the strength of bibliographic coupling between countries. Thicker lines represent stronger connections, meaning that the countries share a higher number of references in their publications. This figure is created by the authors of this study using the tools Biblioshiny and VOSviewer.

Figure 7 illustrates the bibliographic coupling between countries in the field of e-commerce and healthcare research. In this visualization, the size of the nodes represents the number of documents associated with each country, while the thickness of the lines indicates the strength of the bibliographic coupling or the extent to which countries cite the same references in their research. The United States and China emerge as the most prominent nodes, reflecting their substantial research output and influence in this field. These countries are deeply interconnected with others, indicating a high level of shared references and collaboration in e-commerce and healthcare research. The United Kingdom, Canada, and Italy also appear as significant nodes, with strong bibliographic coupling links to various countries, particularly within Europe and North America.

The network reveals that countries like South Korea, Japan, and Singapore are well integrated into the global research landscape, with robust connections to both Western and Asian research hubs. Meanwhile, emerging research contributors like Indonesia, Saudi Arabia, and Egypt are also visible, showing growing participation and citation linkage within the international research community. Overall, this map provides insights into the global research landscape by highlighting the interconnectedness of countries through shared scholarly references, illustrating the collaborative nature of research in e-commerce and healthcare across different regions and nations.

Discussion

The integration of e-commerce and healthcare represents a rapidly growing research area, driven by the increasing use of digital technologies to enhance healthcare delivery across the globe. This study, covering the period from 1993 to 2024, presents a comprehensive bibliometric analysis to understand the evolution of this field, key trends, and major contributors. The analysis reveals a continuous increase in scientific production, particularly after 2019, with a peak in 2021. This surge can largely be attributed to the COVID-19 pandemic, which accelerated the adoption of e-commerce solutions in healthcare through the need for enhanced distancing measures and the demand for digital health services. The slight decline in publications following 2021 likely indicates a stabilization in research activity post-pandemic, with interest in the field persisting but at a more moderated pace.

The thematic evolution observed in this study, as depicted in the trend topics and thematic map, highlights the dynamic nature of research at the intersection of e-commerce and healthcare. Early research predominantly focused on foundational topics such as "e-healthcare" and "telemedicine," which provided the groundwork for more advanced areas like "cloud computing," "big data," and "artificial intelligence." Recently, topics like "blockchain" and "COVID-19" have gained prominence, demonstrating the field's responsiveness to both technological advancements and global circumstances. This evolution underscores the adaptability of research in healthcare e-commerce, where emerging technologies and external events continually shape the discourse.

The analysis identifies China, the United States, and India as the leading contributors to this research field, accounting for a significant portion of total publications. The dominance of these countries is likely linked to substantial investments in digital health infrastructure and the large populations driving the need for scalable e-commerce solutions in healthcare. These countries also exhibit strong international collaboration, as evidenced by the co-authorship and bibliographic coupling analyses. For instance, China’s extensive collaborations with the United States, the United Kingdom, and several European nations highlight the global dimensions of research in this domain. These collaborative efforts play a critical role in advancing innovation and knowledge sharing, which are essential for developing e-commerce applications tailored to diverse healthcare settings.

In terms of key contributors, authors like Liu Y, Zhang X, and Zhang Y have emerged as leading figures in this field, with their research playing a central role in advancing e-commerce in healthcare. The most relevant sources, including Lecture Notes in Computer Science and Proceedings of the International Conference on Electronic Business, reflect the interdisciplinary nature of this research, bridging fields such as technology, healthcare, and business. These sources serve as crucial platforms for disseminating state-of-the-art findings and driving scholarly discourse.

The thematic map analysis provides further insights into the structure of research in this area. Well-developed "Motor Themes" such as "machine learning" and "artificial intelligence" are central to innovation in healthcare e-commerce, underscoring their crucial role in shaping the future of the field. In contrast, themes like "e-health" and "consumer behavior," though highly relevant, appear underdeveloped, suggesting potential areas for future research. Additionally, topics within the Niche Themes quadrant, such as "personalization" and "usability," are highly specialized but less connected to core themes, indicating that further exploration could bridge these areas with more central research themes.

Relating these findings back to the study's objectives, the identified trends in scientific production, author contributions, and global collaboration patterns all align with the broader aim of providing a comprehensive overview of the research landscape. The surge in publications and the prominence of themes like AI and telemedicine reinforce the importance of technological innovation in this field. Furthermore, the strong international collaboration patterns, particularly among leading countries, demonstrate the global effort to develop e-commerce solutions that can address healthcare challenges across different contexts.

In conclusion, this study offers a detailed overview of the evolving field of e-commerce in healthcare, highlighting key trends, contributors, and collaborative networks. The results not only provide insights into how emerging technologies are transforming healthcare delivery but also suggest areas for further exploration, particularly in underdeveloped yet central themes such as e-health and consumer behavior. As this field continues to grow, international collaboration and interdisciplinary research will be pivotal in driving future innovations and shaping the policy landscape for e-commerce applications in healthcare.

Some of the existing studies highlight both the consistencies and divergences between the findings of the present study and the broader literature. The observed surge in e-commerce research within the healthcare sector, particularly during and after the COVID-19 pandemic, parallels earlier studies that emphasized the critical role of digital health technologies during global health crises. For instance, Shahzad et al. documented a significant rise in e-commerce adoption in the Malaysian healthcare sector, driven by factors such as organizational readiness and supply chain integration, which aligns with the upward trend in publications identified in the current study during the same period [3]. Similarly, research on consumer satisfaction within healthcare e-commerce, like that conducted by Chatterjee et al. has underscored the increasing importance of machine learning and sentiment analysis in capturing patient experiences. This corresponds with the emergence of "artificial intelligence" as a key theme in the present study [32]. These comparisons highlight the ongoing evolution of research themes within the intersection of e-commerce and healthcare, reinforcing the findings of the current study while also indicating potential areas for further exploration, particularly in the integration of emerging technologies with healthcare services.

Practical Implications and Future Directions

The findings of this study have some implications for researchers and practitioners concerned with e-commerce and healthcare. First, while the growth rate seems stable, there may be maturity in the research field and an indicator may suggest there is currently a strong base to support further studies. On the other hand, thematic analysis suggests that themes, particularly those found within the basic and niche themes quadrants, still warrant research to better develop e-commerce in such a way that it would jumpstart its potential toward meeting the unique challenges within the health sector.

New technologies and events, like the COVID-19 pandemic, have transformed the research landscape in ways that clearly call for continuous adaptability of the research approach in the future. Future studies should continue to investigate how blockchain and artificial intelligence can be used to enhance healthcare delivery and how specific innovations may be implemented within the context of existing e-commerce structures.

This study provides a comprehensive overview of the e-commerce and healthcare research landscape, highlighting key trends, contributors, and potential areas for future exploration. As the field continues to evolve, it will be essential for researchers to maintain a multidisciplinary approach, leveraging insights from various domains to address the complex challenges at the intersection of e-commerce and healthcare.

This study relies solely on the Scopus database, which may exclude relevant studies indexed in other databases. Additionally, the analysis is predominantly quantitative, focusing on trends and patterns, without delving into the qualitative aspects of the research. Future studies could benefit from using multiple databases and incorporating qualitative approaches for a more comprehensive view.

## Conclusions

This study provides a comprehensive landscape of the evolving e-commerce and healthcare research field, analyzing 1,792 documents from 1993 to 2024. The research highlights a steady growth in scholarly output, with a notable surge in activity beginning in 2019, largely driven by the global COVID-19 pandemic. Leading contributors to this research include countries like China, the United States, and India, reflecting their significant role in integrating digital commerce with health systems on a global scale. Strong international collaboration among these and other major research hubs has enriched the diversity of the literature and fueled advancements in this interdisciplinary field. Thematic and network analyses emphasize the critical role of emerging technologies, particularly artificial intelligence, blockchain, and telemedicine, in shaping the future of e-commerce in healthcare. These technologies, coupled with the impact of the COVID-19 pandemic, have accelerated the need for innovative digital health solutions.

In summarizing these key findings, it is clear that international collaboration and technological innovation will continue to drive future research in this area. Researchers should focus on addressing gaps in foundational themes, such as e-health and consumer behavior, and exploring how these emerging technologies can be leveraged to solve pressing challenges like data security, privacy, and the scalability of healthcare solutions. These insights serve as a valuable resource for guiding future research, policy development, and industry innovation, helping stakeholders navigate the rapid changes in the intersection of e-commerce and healthcare.
